# SNAIL *vs* vitamin D receptor expression in colon cancer: therapeutics implications

**DOI:** 10.1038/sj.bjc.6602484

**Published:** 2005-03-15

**Authors:** M J Larriba, A Muñoz

**Affiliations:** 1 Instituto de Investigaciones Biomédicas ‘Alberto Sols’, Arturo Duperier, 4, Consejo Superior de Investigaciones Científicas-Universidad Autónoma de Madrid, E-28029 Madrid, Spain

**Keywords:** SNAIL, vitamin D, VDR, colon cancer, therapy, E-cadherin

## Abstract

Vitamin D analogues with reduced hypercalcemic activity are under clinical investigation for use against colon cancer and other neoplasias. However, only a subset of patients responds to this therapy, most probably due to loss of vitamin D receptor (VDR) expression during tumour progression. Recent data show that SNAIL transcription factor represses *VDR* expression, and thus abolishes the antiproliferative and prodifferentiation effects of VDR ligands in cultured cancer cells and their antitumour action in xenografted mice. Accordingly, upregulation of *SNAIL* in human colon tumours associates with downregulation of *VDR*. These findings suggest that *SNAIL* may be associated with loss of responsiveness to vitamin D analogues and may thus be used as an indicator of patients who are unlikely to respond to this therapy.

Colorectal cancer is the most frequent neoplasia and the second leading cause of cancer death in Europe, with 218 000 new cases diagnosed each year and an overall rate of 58.1 cases per 100 000 person-years (Ferlay *et al*, 1999). By sex, it constitutes the second most frequent tumour in women after breast cancer and the third in men after lung and prostate tumours. Surgical resection is the therapy of choice for localised tumours, which is curative at an early stage of the disease. However, there is no satisfactory treatment for advanced colon cancer despite the improvement in systemic therapy and radiotherapy in the last decades.

Vitamin D intake and sunlight exposure show a protective effect against colorectal carcinogenesis (Peters *et al*, 2001, and refs. therein). A recent study suggests that vitamin D and calcium supplementation act together to reduce colorectal adenoma risk (Grau *et al*, 2003). In line with epidemiological data, 1*α*,25-dihydroxyvitamin D3 (1,25(OH)_2_D_3_, Calcitriol), the most active metabolite of vitamin D, has antiproliferative, proapoptotic and prodifferentiation effects on many tumour cells *in vitro* (Figure 1), including cells of the large intestine, in addition to its classical role regulating calcium and phosphate homeostasis and bone biology (Hansen *et al*, 2001; Lamprecht and Lipkin, 2003). Remarkably, some vitamin D analogues enhance the cytotoxic effects of chemotherapeutic drugs and radiotherapy.

The clinical use of 1,25(OH)_2_D_3_ is hampered by its hypercalcemic effect. For this reason, a number of 1,25(OH)_2_D_3_ analogues (deltanoids) with variable biological activity and reduced calcemic properties have been synthesised (Carlberg and Mouriño, 2003). Several of them show promising anticancer action in preclinical studies and are currently in clinical trials (Hansen *et al*, 2001). The use of vitamin D analogues for prevention or therapy in cancer patients is still in its infancy. Few Phase I and II studies have been reported, in general showing acceptable toxicity but activity only in a subset of patients.

## 

### 1,25(OH)_2_D_3_ synthesis and action

Dietary vitamin D is scarce, and most vitamin D_3_ is synthesised in the skin by the action of sunlight. Ultraviolet-B radiation converts 7-dehydrocholesterol to previtamin D_3_, which thermoisomerises to vitamin D_3_. This is subjected to two consecutive hydroxylation reactions: first in the liver by vitamin D_3_ 25-hydroxylase (CYP27A1) and then in the kidney by 25-hydroxyvitamin D_3_-1*α*-hydroxylase (CYP27B1) to render 1,25(OH)_2_D_3_. Recently, 25-hydroxyvitamin D_3_-1*α*-hydroxylase has also been found to be expressed by osteoblasts, keratinocytes, chondrocytes, macrophages and several epithelia including the intestinal (MacDonald *et al*, 2001). 1,25(OH)_2_D_3_ is converted to the much less active 1,24,25(OH)_3_D_3_ compound by the 24-hydroxylase (CYP24) (MacDonald *et al*, 2001).

Vitamin D receptor (VDR) is a ligand-regulated transcription factor that mediates most, if not all, the biological effects of 1,25(OH)_2_D_3_ (MacDonald *et al*, 2001). Most 1,25(OH)_2_D_3_ analogues act also via VDR, although some have effects unrelated to VDR binding such as CYP24 enzyme inhibition (Posner *et al*, 2004). For years considered to be exclusively nuclear, VDR is now believed to shuttle constantly between nucleus and cytoplasm; ligand binding and its interaction with the retinoid X receptor (RXR) increase nuclear accumulation (Prüfer and Barsony, 2002). Unliganded VDR binds transcriptional corepressors such as SMRT, N-CoR or Alien, actively repressing target genes. Hormone binding induces a conformational change in VDR that increases the formation of VDR-RXR heterodimers and DNA binding, and causes corepressor release. Subsequently, VDR binds coactivators (SRC-1, ACTR, GRIP-1 and SKIP, among others) and interacts with the multiprotein DRIP (vitamin-D-receptor-interacting protein) and chromatin remodeller complexes, which finally leads to activation of genes mediating most hormone actions (MacDonald *et al*, 2001). Furthermore, VDR is phosphorylated at several residues by different kinases, which contribute to the regulation of its localisation and function in ways that remain to be understood.

In addition to the genomic pathway involving nuclear VDR, a nongenomic pathway of 1,25(OH)_2_D_3_ action has been postulated (Figure 1). This latter pathway is characterised by rapid, gene expression-independent effects such as changes in ion levels or the activity of some kinases, which might be mediated by membrane or cytosolic VDR or by other unknown receptors (Lamprecht and Lipkin, 2003, and references therein).

### 1,25(OH)_2_D_3_ and colon cancer

The predominant effect of 1,25(OH)_2_D_3_, be it antiproliferative, proapoptotic or prodifferentiation, depends on the cell type, and is supposedly based on the set of genes regulated in each particular system. Cell-cycle arrest may result from the induction of cyclin-dependent kinase inhibitors such as p21^CIP^ and p27^KIP^ and the repression of cyclin D1, whereas the activation of *BAX* and *BAK* and the inhibition of *BCL2* and *IAP* contribute to apoptotic induction (Figure 1) (Lamprecht and Lipkin, 2003).

Mutation of *APC* (*adenomatous polyposis coli*) tumour suppressor gene or, less frequently, of *CTNNB1* encoding *β*-catenin, a component of adherens junctions involved in intercellular adhesion, is a common early alteration in human colon cancer (Giles *et al*, 2003). These mutations, or stimulation of the signaling pathway by Wnt extracellular factors, prevent *β*-catenin protein degradation by a complex involving APC, Axin and the protein kinases GSK-3*β* and CKI*α*, causing its accumulation in the cytosol and translocation into the nucleus (Giles *et al*, 2003). In the nucleus, *β*-catenin forms complexes with the TCF/LEF family of transcriptional regulators leading to the activation of genes involved in proliferation and invasiveness, thus promoting colon cancer progression (Giles *et al*, 2003 and references therein).

A study by our group has revealed that 1,25(OH)_2_D_3_ and several nonhypercalcemic analogues promote the differentiation and inhibit the proliferation of human colon cancer cells expressing high VDR levels (SW480-ADH) through the induction of E-cadherin and other adhesion proteins and the blockade of *β*-catenin transcriptional activity (Pálmer *et al*, 2001) (Figure 1). As a result, 1,25(OH)_2_D_3_ inhibits the expression of *β*-catenin target genes such as c-*MYC, TCF1* or *CD44* (Pálmer *et al*, 2001). In total, 200 1,25(OH)_2_D_3_ target genes have been reported in SW480-ADH cells: they are involved in different aspects of cell biology and support the beneficial effect of the hormone on cell phenotype and growth (Pálmer *et al*, 2003). In summary, 1,25(OH)_2_D_3_ regulates many genes either directly, through VDR binding to their regulatory regions, or indirectly, via intermediate genes or by affecting other pathways, such as Wnt/*β*-catenin, that are related to its antitumoral action.

### VDR and colon cancer

In the human colon, normal epithelial cells and some cancer cells express VDR (Kállay *et al*, 2002). Furthermore, some colon cancer cells express 25-hydroxyvitamin D_3_-1*α*-hydroxylase and can thus synthesise 1,25(OH)_2_D_3_ from its precursor (Cross *et al*, 2001). This suggests that the VDR expression observed in low-grade tumours and the tumour-localised production of this hormone could be an autocrine/paracrine means to control tumour progression. Concordantly, a high level of VDR expression is associated with a favourable prognosis in colorectal cancer (Cross *et al*, 1996; Evans *et al*, 1998). Vitamin D receptor expression is enhanced during early stages of colon cancer but, analogously to oestrogen and progesterone receptors in breast cancer, it is downregulated during late colon cancer progression (Sheinin *et al*, 2000; Cross *et al*, 2001), causing ligand unresponsiveness and, possibly, failure of therapy with vitamin D analogues (Figure 2). Some studies indicate that VDR expression decreases in high-grade carcinomas to levels found in normal mucosa (Sheinin *et al*, 2000; Cross *et al*, 2001; Kállay *et al*, 2002), while others found diminished VDR expression already in low- and intermediate-grade tumours and a decrease below normal mucosa levels in high-grade carcinomas (Pálmer *et al*, 2004) (Figure 2).

These data suggest that VDR-deficient mice (whose phenotype closely resembles that of human with vitamin D-dependent rickets type II) may be prone to develop colon cancer. However, these animals display hyperproliferation and oxidative stress in the distal colon, but not elevated rates of spontaneous colon cancer (Kállay *et al*, 2001). Likewise, mice lacking 25-hydroxyvitamin D_3_-1*α*-hydroxylase show similar abnormalities to humans with vitamin D-dependent rickets type I, while no changes in intestines or tumour development have been reported (Bikle *et al*, 2004).

Several *VDR* polymorphisms have been described. However, most *VDR* polymorphisms do not result in structural alteration in the VDR protein and are of unknown functional significance and unrelated to adenoma recurrence, and furthermore do not modify the associations with vitamin D or calcium (Peters *et al*, 2001; Grau *et al*, 2003; Sutton and MacDonald, 2003; Harris and Go, 2004).

### SNAIL: biological effects and regulation

SNAIL is a zinc-finger transcription factor expressed in migratory processes during embryonic development that has recently been implicated in cancer (Nieto, 2002). *SNAIL* upregulation in epithelial cells induces the acquisition of cell movement and invasiveness, at least in part due to the repression of E-cadherin expression (Batlle *et al*, 2000; Cano *et al*, 2000). In the E-cadherin gene promoter, SNAIL binds to regions that contain the consensus core sequence (CAGGTC), a motif that is a subset of the E-box (CANNTG), the conserved binding site of basic helix-loop-helix transcription factors. The acquisition of the invasive phenotype is linked to SNAIL upregulation and concomitant E-cadherin repression in gastric, melanoma, breast and hepatocellular cancer cells. Either directly or indirectly, SNAIL also downregulates other genes encoding adhesion proteins such as several claudins, occludin and ZO-1, and also those for MUC-1 and aromatase, while it upregulates several metalloproteinases in different cell types. Additionally, SNAIL attenuates cell proliferation and confers resistance to apoptosis in breast cancer cells (Vega *et al*, 2004). Supporting a role of SNAIL in colon tumorigenesis, downregulation of SNAIL suppresses both total tumour number and incidence of large tumours in APC^min^ mice (Roy *et al*, 2004). Studies performed with human and murine *SNAIL* gene promoters have evidenced the dependence of the transcription of this gene on the activity of signalling pathways involving MEK1/2, PI3K/Akt and NF*κ*B/p65 (Peinado *et al*, 2003; Barberá *et al*, 2004). In mammary epithelial cells, *SNAIL* is repressed by *MTA-3*, a gene induced by oestrogen.

### Relation between *SNAIL* and *VDR* expression

Our group observed that in a panel of human colon cancer cell lines, high *SNAIL* expression associates with undifferentiation and low *VDR* and E-cadherin expression, and *vice versa*. Subsequent work revealed that SNAIL protein binds to and represses (50%) the human *VDR* gene promoter (Pálmer *et al*, 2004). As a result, SNAIL abolishes the induction by 1,25(OH)_2_D_3_ of E-cadherin and other target genes (Figure 1). Moreover, SNAIL-overexpressing human colon cancer cells became resistant to growth inhibition by the vitamin D analogue EB1089 when they were injected subcutaneously in immune-deficient mice. In humans, we have found increased *SNAIL* RNA expression in tumour *vs* normal tissue (22 out of 32 patients), and a significant inverse correlation between the expression of *SNAIL* and that of *VDR* and E-cadherin (Pálmer *et al*, 2004). A later study of a larger series of patients confirmed this relation, and showed that loss of *VDR* RNA is linked to poor differentiation (F Bonilla, personal communication). These data indicate that the loss of *VDR* RNA expression during colon cancer progression is probably related to *SNAIL* upregulation, and therefore that high levels of SNAIL may be responsible for the failure of therapy with vitamin D analogues in patients with this and perhaps other neoplasias (Figure 2). In contrast to our data, *SNAIL* RNA expression was only detected in a low proportion of human colon tumours in another study (Rosivatz *et al*, 2004). The reasons for this discrepancy are unclear.

Loss of E-cadherin has been causally associated to the transition of adenoma to carcinoma and the acquisition of migration capacity. As stated above, VDR is also lost at late stages of colon cancer progression, impairing the induction of E-cadherin by 1,25(OH)_2_D_3_. Concordantly with these and our results, we propose that SNAIL promotes the acquisition of invasive behaviour via the repression of both *VDR* and E-cadherin genes. The repression of *VDR* by SNAIL is particularly relevant due to the protective action of 1,25(OH)_2_D_3_ and analogues on colon carcinogenesis, and constitutes a second mechanism for the efficient repression of E-cadherin. We have not found regulation of *SNAIL* RNA expression in colon cancer cells by 1,25(OH)_2_D_3_. Subcellular localisation and degradation of SNAIL protein is regulated by phosphorylation (Zhou *et al*, 2004) but, likewise, 1,25(OH)_2_D_3_ does not change SNAIL protein localisation in SW480-ADH cells.

During development, SNAIL expression is linked to cell deadhesion and migration processes, but it is downregulated when the cells have reached their destination. In human breast cancer metastasis E-cadherin is re-expressed (Bukholm *et al*, 2000), suggesting that SNAIL might be downregulated. Whether this occurs in colon metastasis remains to be elucidated. Likewise, no data are available on *VDR* expression in metastasis. The possibility that SNAIL upregulation reported in melanoma, gastric or other cancers may also cause VDR downregulation in these neoplasias remains unexplored. The *VDR* gene is subjected to complex transcriptional regulation: Wilm's tumour suppressor, Zeb-1, Cdx-2 and Sp1 transcription factors as well as protein kinase A induce *VDR* expression. At least during tumorigenesis, SNAIL seems to overcome these factors.

### Therapeutic implication of the repression of *VDR* by *SNAIL*

Only a subset of cancer patients treated with vitamin D analogues respond to therapy. As with other therapies, it is important to select those patients that are likely to respond. An example is the selection of breast cancer patients with high HER2/Neu expression as candidates for treatment with anti-HER2/Neu antibodies. Our data indicate that colon cancer patients with *SNAIL* upregulation express low VDR and are thus likely to be poor responders to vitamin D analogues. As *SNAIL* upregulation seems to be linked to the acquisition of the migratory/invasive phenotype, which correlates with the metastatic potential, patients with low-grade colon cancers are preferential candidates for this therapy (Figure 2). In cases of acceptable toxicities, they may also be useful as chemopreventive agents for high-risk patients. Our studies show that the balance between *VDR* and *SNAIL* expression plays an important role in the control of cell fate during colon cancer progression, and emphasise the need to analyse *VDR* and/or *SNAIL* expression in colon cancers in order to select patients suitable for therapy with vitamin D analogues. An attractive, albeit unexplored, possibility is the study of *SNAIL* or *VDR* RNA levels in blood, a strategy used for cytokeratin 19, carcinoembryonic antigen and *β*-catenin in colorectal cancer (Silva *et al*, 2002) and other genes in different neoplasias. If upregulation of *SNAIL* or downregulation of *VDR* in tumours could be monitored by RT–PCR analysis of circulating RNA levels, this would constitute a valuable tool for the selection of candidates for therapy with vitamin D analogues.

## CONCLUSIONS

1,25(OH)_2_D_3_ has profound effects on gene expression, inducing differentiation and inhibiting proliferation of colon cancer cells. Preclinical and epidemiological data suggest that vitamin D analogues may be useful for the prevention and treatment of colorectal cancer. To date, however, therapy with these compounds has benefited only a limited number of cancer patients. The SNAIL transcription factor inhibits the expression of genes encoding adhesive proteins and causes the acquisition of migratory and invasive phenotype of epithelial cells, and is thus considered an important factor of malignancy. Recent data have shown that SNAIL represses *VDR* gene expression in cultured human colon cancer cells, leading to hormone unresponsiveness *in vitro* and *in vivo*. Since *SNAIL* is upregulated during human colon cancer progression, which is associated with reduced VDR expression, the analysis of *SNAIL* expression may help to select patients suitable for therapy with vitamin D analogues.

## Figures and Tables

**Figure 1:**
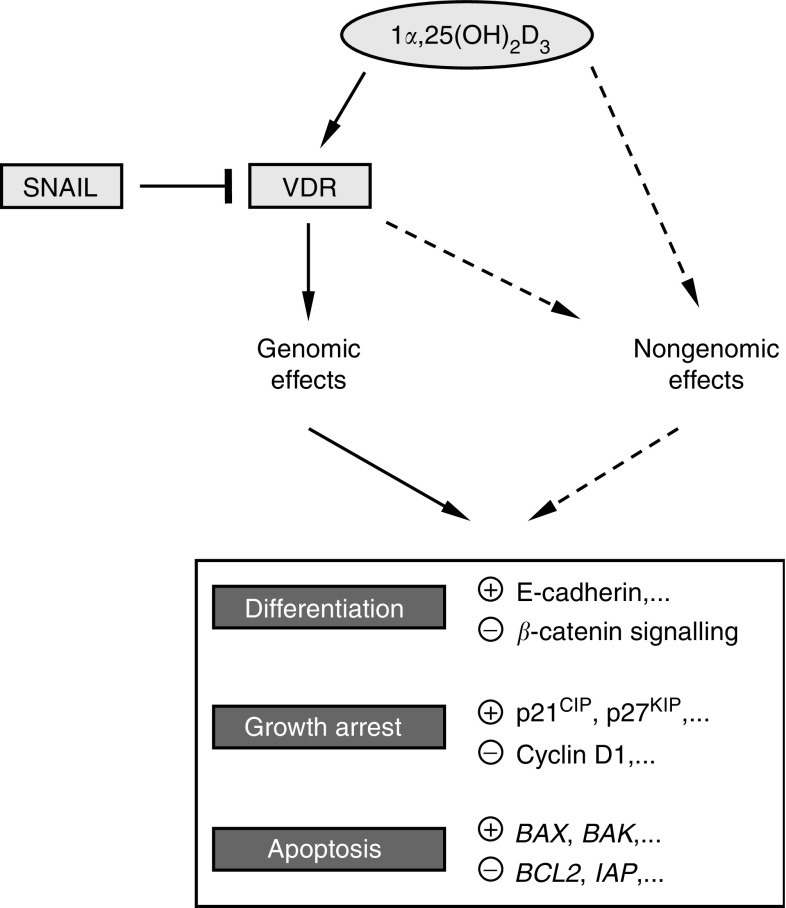
Effects of 1,25(OH)_2_D_3_ in colon cancer cells. SNAIL inhibits 1,25(OH)_2_D_3_ action by repressing *VDR* gene expression. Discontinuous lanes refer to not confirmed processes. 1,25(OH)_2_D_3_ inhibits *β*-catenin signalling (Pálmer *et al*, 2001) and up- or down-regulates numerous genes, directly or indirectly (Lamprecht and Lipkin, 2003; Pálmer *et al*, 2003).

**Figure 2:**
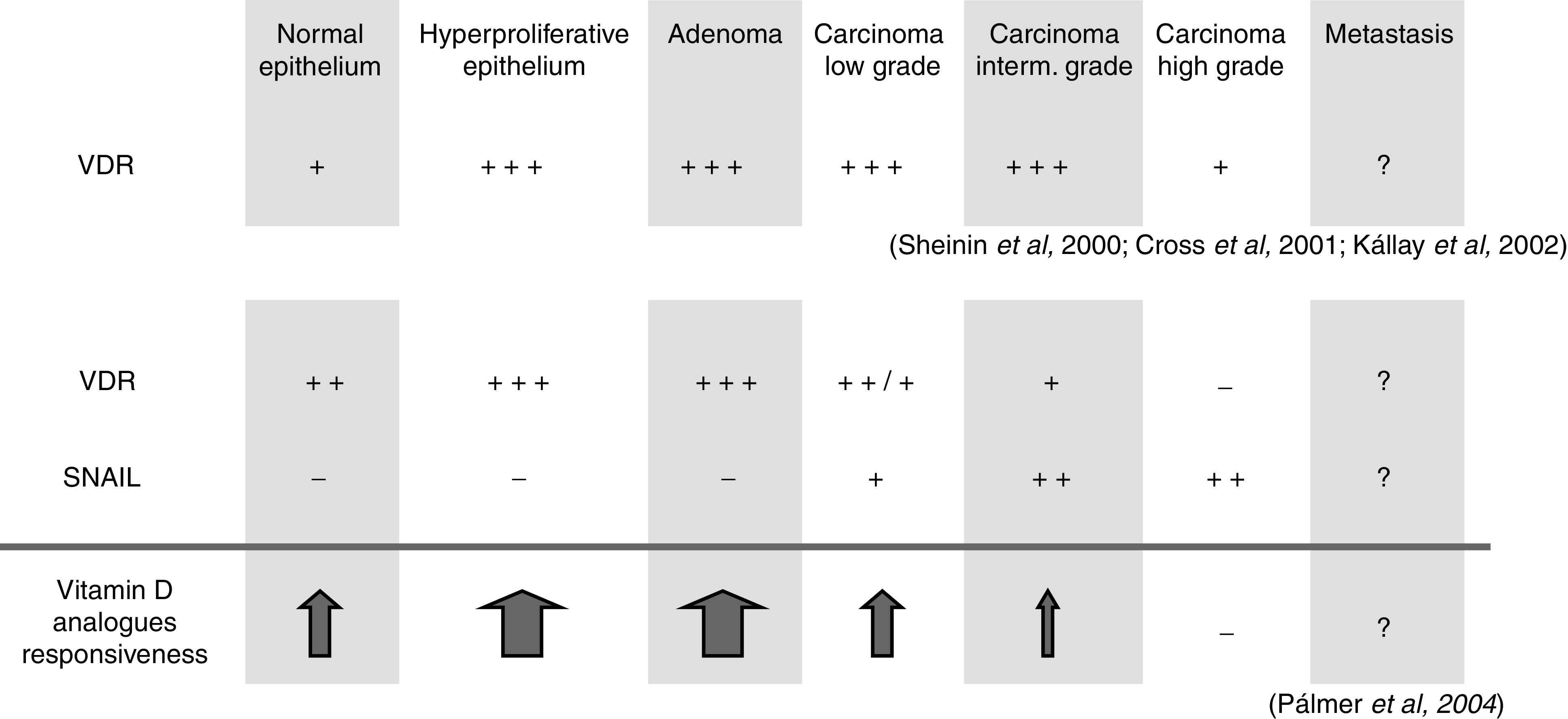
Opposite pattern of expression of *VDR* and *SNAIL* during human colon cancer progression. As indicated, downregulation of *VDR* has been found in some studies only in high-grade carcinomas, whereas in other studies it is observed already in low- and intermediate-grade tumours. These data indicate that the efficacy of therapy with 1,25(OH)_2_D_3_ analogues is lost at late steps of tumour progression.
